# Triboelectric Energy Harvesting Response of Different Polymer-Based Materials

**DOI:** 10.3390/ma13214980

**Published:** 2020-11-05

**Authors:** Tiago Rodrigues-Marinho, Nelson Castro, Vitor Correia, Pedro Costa, Senentxu Lanceros-Méndez

**Affiliations:** 1 Center of Physics, Campus Gualtar, University of Minho, 4710-057 Braga, Portugal; tiagomarinho.fis@gmail.com (T.R.-M.); eng.v.correia@gmail.com (V.C.); 2 BCMaterials, Basque Center for Materials, Applications and Nanostructures, UPV/EHU Science Park, 48940 Leioa, Spain; nelsonjfcastro@gmail.com (N.C.); senentxu.lanceros@bcmaterials.net (S.L.-M.); 3 Algoritmi Research Center, University of Minho, 4800-058 Guimarães, Portugal; 4 Institute for Polymers and Composites IPC/i3N, University of Minho, 4800-058 Guimarães, Portugal; 5 IKERBASQUE, Basque Foundation for Science, 48009 Bilbao, Spain

**Keywords:** triboelectric effect, polymer and composites, energy harvesting, low-power devices

## Abstract

Energy harvesting systems for low-power devices are increasingly being a requirement within the context of the Internet of Things and, in particular, for self-powered sensors in remote or inaccessible locations. Triboelectric nanogenerators are a suitable approach for harvesting environmental mechanical energy otherwise wasted in nature. This work reports on the evaluation of the output power of different polymer and polymer composites, by using the triboelectric contact-separation systems (10 N of force followed by 5 cm of separation per cycle). Different materials were used as positive (Mica, polyamide (PA66) and styrene/ethylene-butadiene/styrene (SEBS)) and negative (polyvinylidene fluoride (PVDF), polyurethane (PU), polypropylene (PP) and Kapton) charge materials. The obtained output power ranges from 0.2 to 5.9 mW, depending on the pair of materials, for an active area of 46.4 cm^2^. The highest response was obtained for Mica with PVDF composites with 30 wt.% of barium titanate (BT) and PA66 with PU pairs. A simple application has been developed based on vertical contact-separation mode, able to power up light emission diodes (LEDs) with around 30 cycles to charge a capacitor. Further, the capacitor can be charged in one triboelectric cycle if an area of 0.14 m^2^ is used.

## 1. Introduction

The world is experiencing a rapid revolution in the mode in which energy is being produced and consumed in daily life and industry [1,2]. Conventional ways to produce energy need to adapt to the environmental needs and concerns related to sustainability and, on the other hand, the energy consumption paradigm has also been strongly changing in the last decade based on increased mobility [2]. Thus, cell phones, tablets or related gadgets are common and ubiquitous nowadays. Hydroelectric energy generation remains the pillar of renewable energies [3,4], wind and solar energy generation are becoming increasingly important in the energy generation share [3].

In the last decade, with the fast development of the Internet of Things (IoT) [5] and portable electronics [5], the demand for a sustainable and environmentally friendly portable power supply is becoming very significant. In this context, energy generation systems are an interesting option for portable technologies, though still show low power output and, therefore, a low range of applications [3]. Piezoelectric, pyroelectric or thermoelectric energy generation are among the most studied technologies, though efficiency and power output [6] is limited compared to electrostatic or triboelectric energy harvesting devices [6].

Piezoelectric [3,7], electrostatic [8], electromagnetic [9] and triboelectric [10,11,12] energy harvesting technologies rely on the use of wasted mechanical energy (wind, wave, vibrations and even body movements) to produce electricity. These mechanical harvesters can be based on polymer and polymer composite materials, offering unique properties, such as lightweight and flexibility, combined with easy integration and environmentally friendly processability [3].

The power density per area of triboelectric devices is the largest among the aforementioned systems, reaching powers as high as 500 W·m^−2^ and an energy conversion efficiency of 70% has been demonstrated [9,13], further they are lightweight and cost-effective [13]. Compared with piezoelectric devices, triboelectricity can be more suited for environmentally friendly energy production for portable devices [3], low-power devices in remote or inaccessible places [14] or for needed IoT network of sensors [14].

Another advantage of the triboelectric phenomenon is the wide range of materials that can be used in the distinct triboelectric mechanical modes: contact-separation, lateral sliding, single electrode and free-standing triboelectric layer mode [5,14]. Being a process that can be carried out entirely with polymers and the corresponding composites, the overall properties of the materials can be tailored for each specific application, including dimensions, geometry and optical transparency. The triboelectric power output can also be strongly improved by tailoring the intrinsic properties of the polymers by synthesis and functionalization [15] or by reinforcing with high-dielectric or other functional fillers [9]. Further, geometrical dimensions (mainly the thickness) and roughness of the materials can also be designed to maximize the generated energy.

Literature reports different materials and order within the triboelectric series [9,16,17] in terms of relative triboelectric charge providing/receiving characteristics. The most interesting triboelectric materials are those with easy lose and gain electrons when in contact, leading to higher charge density between two different materials [18]. The most common materials in the literature are several polymers, but also some metals and crystalline materials [17,18,19,20]. Positive (losing electrons) materials include Mica (silicate) and glass, polyamide 6-6 (PA66) and polyamide 11 (PA11), polyethylene (PE) silk, aluminum, paper and polyvinylidene fluoride (PVDF) [9,12,16,17,20]. Negative materials (gaining electrons) include polytetrafluoroethylene (PTFE or Teflon), polyvinyl chloride (PVC), polyimide (Kapton), polystyrene, rubber-like or polyurethane (PU), among others [12,17,18,19,20]. The surface charge density of the triboelectric mode depends on the pair of the selected materials and range change from some nC.m^−2^ to mC.m^−2^ [18], being higher for polymeric pairs compared to metallic ones.

Triboelectric energy generation optimization includes, in addition to materials selection, tailoring the functional properties of the material reinforcing with dielectric fillers, selection of the triboelectric mode among contact-separation, sliding, single electrode or freestanding modes and, finally, the electronic circuit to harvest the electrical energy [14,18,19,20,21]. Ceramic or low amounts of conductive nanofillers [19,20] can be used to improve the dielectric properties and, correspondingly, the triboelectric performance of the materials. Triboelectric modes and electronic circuits allow the combination of two distinct modes or even, combine the piezoelectric and thermoelectric effects [21,22].

In this work, the triboelectric properties of the different materials, mostly polymers and polymer composites are evaluated. Pairs of materials in different places in the triboelectric series are evaluated, together with materials prepared by different technologies (solvent based, hot pressing and commercial materials). Finally, high-dielectric ceramic nanomaterial (barium titanate- BT) have been used to improve the dielectric and, consequently, the triboelectric power output of one of the materials. In this way, a complete set of materials and processing conditions are considered, allowing triboelectric output understanding, material selection and tailoring for specific applications.

## 2. Experimental

### 2.1. Materials

Thermoplastic elastomer styrene-ethylene/butylene-styrene (SEBS) copolymer (Calprene CH-6120, Madrid, Spain) with an ethylene-butylene/styrene ratio of 68/32 was supplied by Dynasol Gestión, S.I. (Madrid, Spain). Commercial polyvinylidene fluoride (PVDF) (Solef 6010), with density of 1.75 g/cm^3^ was supplied by Solvay (Paris, France). The solvent used to process SEBS was cyclopentyl methyl ether (CPME) supplied by Carlo Erba Reagents (Val de Reuil, France) (density of 0.86 g/cm^3^ at 20 °C; boiling point of 106 °C) and for PVDF, it was N,N-dimethylformamide (DMF, 99.5%) from Merck (Darmstadt, Germany).

A commercial sheet of PVDF (PVDF-c) with 1 mm of thickness was obtained from Swami Plast Industries (Gujarat, India) and Mica, Kapton and polyurethane (PU) were obtained from Agar Scientific (Essex, UK), Dupont (Faro, Portugal) and SWM-Engineered (Genk, Belgium), respectively. Polyamide 66 and polypropylene (PP) pellets were purchased from Merck (Sigma-Aldrich, St. Louis, MO, USA).

For the preparation of the polymer composite, barium titanate (BT), particles with an average size of 100 nm and a dielectric constant of 150 were obtained from Merck (Sigma-Aldrich, St. Louis, MO, USA).

### 2.2. Sample Preparation

Three types of different processed materials were investigated: solvent cast films for pristine polymers and polymer composite with BT, hot pressing and commercial polymers in sheet form.

The solvent casting method was similar for SEBS, PVDF and composites with solvent/polymer ratio of 80/20 *v*/*v* using about 1 g of polymer for 5 mL of solvent. For the SEBS dissolution, CMPE was used as the solvent, while DMF was used for the PVDF. Once the corresponding amount of polymer and solvent were added, the mixture was magnetically stirred for 3 h at 30 °C until complete polymer dissolution. For the PVDF composite, the corresponding amount of BT nanoparticles (30 weight percentage (wt.%) to maximize dielectric response were maintaining mechanically flexible films [23]) were homogeneously dispersed in DMF in an ultrasonic bath at 25–35 °C for 2 h, then the PVDF was added and the mixture was magnetically stirred for 3 h at 30 °C.

Thin films were obtained by spreading the mixtures on a clean glass substrate using the doctor blade technique with a 200 µm blade thickness. SEBS samples were dried at 30 °C for 12 h, whereas PVDF samples were melted in an oven at 210 °C for 20 min and recrystallized by cooling down to room temperature, promoting the crystallization of the PVDF in the α-phase and achieving complete solvent evaporation [24,25,26]. The different processed samples are represented in Table 1. The thicknesses of the films after complete evaporation of the solvent ranged from 40 to 60 µm.

The use of solvents was avoided in PA66 and PP, positive and negative triboelectric materials, respectively (Table 1). Both polymers were produced by the hot pressing method where 20 g of polymer pellets were placed in a hot-pressing machine (from Metalgrado LDA, Porto, Portugal) for 15 min at a temperature of 220 °C between two 40 × 40 cm sheets of Teflon. After removing, the film thickness was about 1 ± 0.1 mm.

Commercial films of different materials, including Mica, PVDF-c, PU and Kapton, with a thickness of about 1 mm, were also used. Samples produced by solvent casting and hot pressing and commercially available materials were also evaluated and compared. In this way, some of the most interesting triboelectric polymers have been comparatively evaluated to understand and optimize triboelectric output.

**Figure 1:**
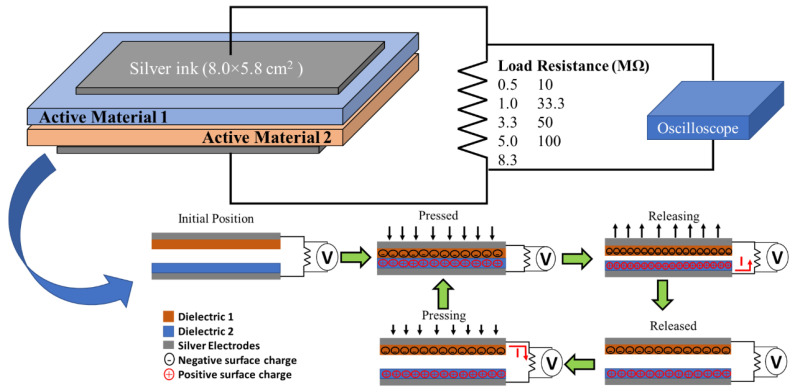
Illustration of the geometry of the samples with the active material, screen-printed silver ink electrodes and their connection to the Picoscope 2205A using load resistances of 0.5, 1, 3.3, 5, 8.3, 10, 33.3, 50 and 100 MΩ, above. Experimental method used for the triboelectric evaluation of the different pair of materials in a contact-separation mode, below. The force applied in each step is about 10 N.

In order to collect the charge provided by the triboelectric effect, conductive silver ink (Electronic 131 paste DT1201, hunan LEED electronics Ink, Zhuzhou, China) was deposited on the outer surface of each material within a home-made screen-printing set-up using a squeegee over the screen placed at 1 mm distance from the substrate. After the printing step, the material and silver ink were dried at 60 °C for 60 min in an oven (Binder E, model 28, Binder, Tuttlingen, Germany). The printed electrode area was 8.0 × 5.8 cm, as shown in Figure 1. The final step of sample preparation was to place the active materials with electrodes on substrate support fabricated by 3D printing (Sigma R19 BCN3D with 20% PLA filling, BCN3D, Barcelona, Spain) with dimensions 10 × 6 × 1 cm (slightly larger than the active materials) to perform the triboelectric measures. To assure good adhesion of the active material to the substrate, double-sided adhesive tape (Tesa 4970, Tesa, Lisboa, Portugal) was used.

Table 1 summarizes the materials used in this work considering the triboelectric series, including the composite with 30 wt.% BT/PVDF composite (30BT/PVDF).

### 2.3. Triboelectric Measurements

The triboelectric mode used to determine the output voltage, current and corresponding output power of the different pairs of materials (Table 1) was the contact-separation mode (Figure 1): a force of 10 N was applied in the contact mode, followed by a separation of 5 cm between the samples in each cycle.

The electrical response was obtained though load resistances of 0.5, 1, 3.3, 5, 8.3, 10, 33.3, 50 and 100 MΩ using a Picoscope 2205A (Picotech, Tyler, TX, USA) with resolution of 8 bit at 200 MS/s.

## 3. Results and Discussion

### 3.1. Triboelectric Output

**Figure 2:**
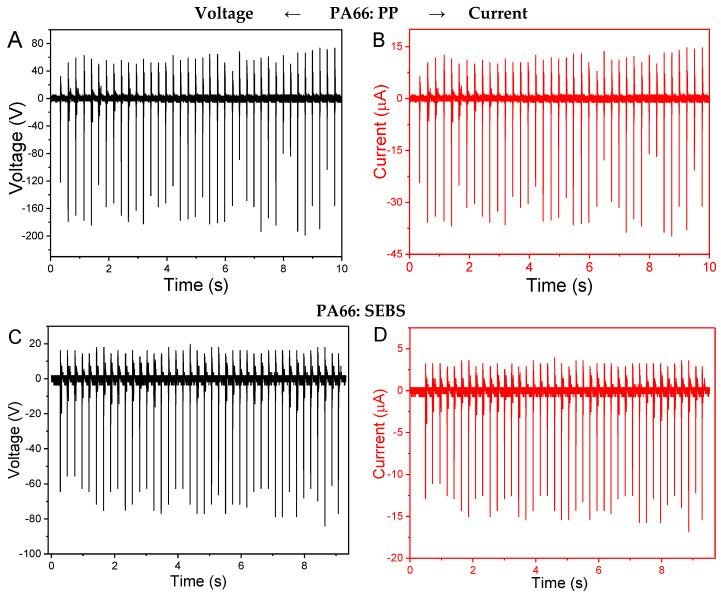

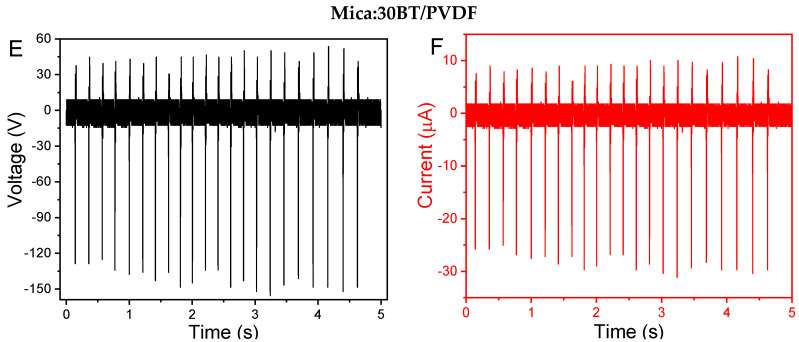
Open-circuit voltage (left) and the short-circuit current (right) measurements for different pairs of polymers. The pairs PA66:PP (**A**,**B**), PA66:SEBS (**C**,**D**) and Mica:30BT/PVDF (**E**,**F**), measured under with a R_L_ = 5 MΩ under constant force (10 N) in contact-separation mode.

The triboelectric voltage and current output of the different pairs of materials represented in Table 1 are shown in Figure 2 for a load resistance of 5 MΩ. The voltage is determined in open-circuit (Figure 2A,C,E) and the current in short-circuit (Figure 2B,D,F) for representative pair of materials, measured with a load resistance (R_L_) of 5 MΩ under a constant force (10 N) in the contact-separation mode.

**Figure 3:**
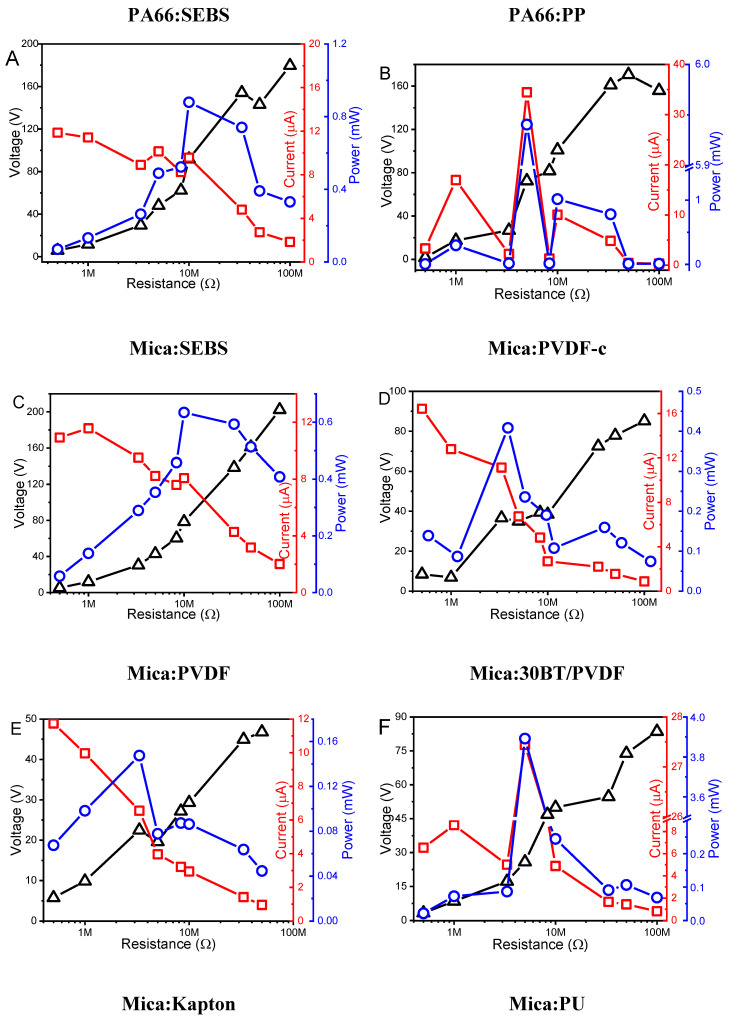

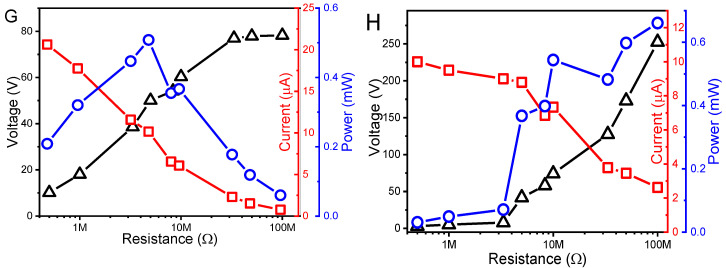
Triboelectric output voltage, current and power as a function of the load resistance for different material pairs: (**A**) PA66:SEBS; (**B**) PA66:PP; (**C**) Mica:SEBS; (**D**) Mica:PVDF-c; (**E**) Mica:PVDF; (**F**) Mica:30BT/PVDF; (**G**) Mica:Kapton and (**H**) Mica:PU.

Among the different pairs of materials measured (Table 1B), Figure 2 illustrates, as representative examples, the triboelectric performance of PA66:PP, PA66:SEBS and Mica:30BT/PVDF. The output voltages per cycle of these pair of materials are between approximately 60 and 150 V (in average for 40 cycles) and the current generated per cycle ranges between 12 to 30 μA, for the same experimental conditions. Further, it is to notice that the 30BT/PVDF sample shows piezoelectric properties (due to the piezoelectric ceramic material) that contribute to a piezoelectric voltage generation in each cycle (mechano-electrical conversion), together with the triboelectric energy generation, as can be observed in Figure 3E,F. It is to notice that the piezoelectric voltage generation is small in comparison to the triboelectric contribution.

Literature reports a wide amplitude of the voltages and currents generated in triboelectric systems, from some volts to thousands of volts [29,30,31,32,33], and current typically ranging up to hundreds of μA [31,32,33,34]. The voltage and current output values obtained in the present work are competitive with the literature, considering the use of pristine materials without any kind of surface treatment.

Thus, the above materials allow us to generate up to 150 V per cycle through the triboelectric effect in contact-separation mode, suitable for low-power devices [22], as it will be demonstrated later.

Based on the representative experimental results shown in Figure 2, the output voltage and current triboelectric output of all materials pairs are shown in Figure 3 and Table 2 as a function of the external load resistance (R_L_) in the range from 0.5 to 100 MΩ. All systems show a similar electrical output response with a maximum output power for R_L_ = 3 to 10 MΩ. Increasing R_L_ leads to an output voltage increase and a decrease of the current, leading to maximum output power (Power (P) = voltage × current) at the interception of these [16].

The power output performance depends on several factors, such as triboelectric charge providing/receiving [11,34] and physical properties of the materials [35,36]. It is shown in the literature that materials further apart in providing/receiving electrons lead to a larger triboelectric output than materials close to each other, which may exchange small amounts of charge [34]. Roughness is also a key factor for triboelectric energy generation. It has been experimentally demonstrated for different polymer-based materials that increasing roughness leads to an increase in the output power of the triboelectric materials [35,36,37].

The triboelectric performance of the PA66:SEBS pair is represented in Figure 3A, showing a P = 0.90 mW at a R_L_ = 5 MΩ. It is to notice that these materials are close in the triboelectric series, but one prevalent factor, surface roughness, also plays a relevant role, as mentioned before. Solvent cast samples present higher roughness than commercial ones, leading to a higher surface area, resulting in improved triboelectric performance. Also, the samples prepared by hot-pressing, such as PA66, show a larger surface roughness and, as a consequence, the PA66:PP pair shows a P = 5.94 mW for R_L_ = 5 MΩ, as is shown in Table 2.

Thus, polyamide was used combined with SEBS and PP with a maximum power of 0.9 and 5.8 mW, respectively. Mica was also used to compare the triboelectric performance of the samples PVDF or PVDF-c, considering the processing method. Both have a maximum power around R_L_ = 3.3 MΩ with the commercial and solvent cast samples reaching the same order of magnitude for the output power, about 0.4 mW instead of 0.2 mW for PVDF-c and PVDF (Figure 3D,E), respectively. A similar performance indicates that the intrinsic properties of the PVDF materials, in particular the large dielectric constant (ε ≈ 6) [38], overcomes the effects related to the manufacturing process and surface roughness variations.

The important role of the dielectric properties in the triboelectric output of the samples is demonstrated by the PVDF composites reinforced with barium titanate. The high dielectric constant of the ceramic nanoparticles (150) embedded into the PVDF matrix (dielectric constant around 6) leads to an increase of dielectric constant with an increase of filler content [39,40] up to ε ≈ 15 for the composite PVDF, increasing the performance of the triboelectric system, as predicted by theoretical models [9,41,42] and experimental measurements [38]. Thus, a maximum power output of 0.2 and 3.9 mW was obtained for neat PVDF and 30BT/PVDF, respectively, as shown in Figure 3F, demonstrating that increasing the dielectric constant of a specific material leads to an increase of its triboelectric output. In conclusion, the roughness and dielectric permittivity of polymers influence the triboelectric performance, being that the dielectric properties are more preponderant in the charges transferred between opposite surfaces.

Commercial pair of materials Mica:Kapton presents a P = 0.5 mW for R_L_ = 5 MΩ, despite being one of the most opposite pairs within the triboelectric series. Contrary to PVDF, surface treatments in the surface (smooth surfaces) of the commercial materials decreases their triboelectric performance. A similar effect can be observed in Mica:PU with P = 0.7 mW. In this case, the output power continues to increase with increasing R_L_, leading to an output voltage that increases up to 250 V. Mica:SEBS, on the other hand, reaches P = 0.6 mW at R_L_ = 10 MΩ (Figure 3C). SEBS with Mica, being similar to Mica:PA66, despite the proximity of these materials in the triboelectric series.

### 3.2. Energy Harvesting Application

**Figure 4:**
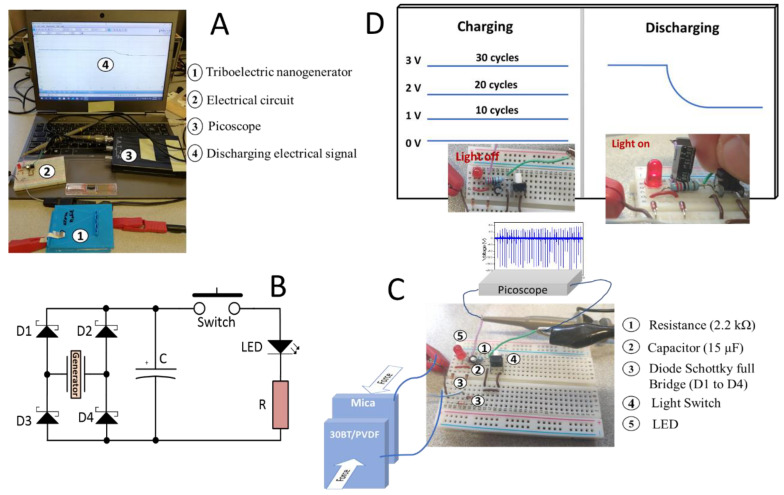
Illustration of the complete setup (**A**) with the pair of materials, detailed electronic circuit scheme (**B**) and Picoscope connected to a laptop. (**C**) Electric circuit for powering the LED and (**D**) charge-discharge cycles using triboelectric materials (PA66:PP or Mica:30BT/PVDF pairs) as nanogenerators.

A simple application was developed by harvesting the triboelectric energy into a capacitor and later powering a LED (Figure 4A). The two material pairs with the largest output powers, PA66:PP and Mica:30BT/PVDF, were used. The triboelectric pairs were connected to an electrical circuit containing 4 diodes in order to transform the AC to DC voltage and charge a capacitor of 15 µF, as illustrated in Figure 4B.

The circuit follows a traditional DO-35 Schottky (D1 to D4) rectifier bridge topology with an output electrolytic capacitor for energy storage, powering a load composed by a manual switch button, the LED and the resistor (Figure 4C). This setup enables the energy to be stored and manually discharge over the load when the voltage level is suitable.

When the capacitor is charged, using a light switch, the LED was lighted on and the respective voltage drop at the capacitor ends was observed (Figure 4C).

By using Mica:30BT/PVDF or PA66:PP pairs it is possible to charge the 15 μF (capacitor with 25 to 30 cycles, the capacitor being able to turn on the LED for a few seconds) (Figure 4D). It is to notice that this is achieved with a small active area of 46.4 × 10^−4^ m^2^ in each material. Thus, by increasing the active area of the materials to 0.14 m^2^, the capacitor could be charged in just one cycle. Thus, implemented in an example, a human walking can generate in a few steps enough energy to power the LEDs, taking into account the weight and area of the shoe.

## 4. Conclusions

The triboelectric effect using a polymers as active materials can be used to harvest energy for low-power devices. This work compares pairs of materials in different places within the triboelectric series, showing that not just the place within the triboelectric series, but also the surface roughness and the dielectric constant play a critical role in determining, to some extent, triboelectric power output. Thus, PVDF composite with higher dielectric constant (2.5 × higher) than pristine polymer generates 10 × and 15 × times larger power (3.89 mW) when compared to commercial or solvent-based PVDF.

Pairs of polymers PA66:PP (P = 5.94 mW) and Mica:30BT/PVDF (P = 3.89 mW) show the larger triboelectric output power among the evaluated materials. Rubber-like material, such as SEBS, present good triboelectric performance with both negative and positive materials (PA66 and Mica).

Finally, it was shown that the generated triboelectric energy can be stored in a 15 μF capacitor and, after 30 cycles, allows the powering of a LED or other low-power application.

## Figures and Tables

**Table 1:** (**A**) Materials within the triboelectric series used in the present work [27,28] and (**B**) pair of materials used in the contact-separation mode triboelectric experiments.

**(A)**			
**Positive**		**Negative**	
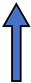	Mica polyamide 6-6 (PA66) styrene-ethylene/butylene-styrene (SEBS)	polyvinylidene fluoride (PVDF) polyurethane (PU) polypropylene (PP) polyimide (Kapton)	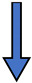
**(B)**			
**Mica**		PVDF polyvinylidene fluoride commercial (PVDF-c) 30 wt.% barium titanate/polyvinylidene fluoride (30BT/PVDF) Kapton PU SEBS	
**PA66**		SEBS PP	

**Table 2:** Summary of the output power, voltage and current for the different systems under evaluation, the larger output powers being for the PA66:PP and the Mica:30BT/PVDF pairs.

Materials	R_L_ (MΩ)	Voltage (V)	Current (µA)	Power (mW)
**PA66:PP**	5	172.5	34.4	5.9
**PA66:SEBS**	5	68.1	13.2	0.9
**Mica:SEBS**	10	78.6	8.1	0.6
**Mica:PVDF-c**	3.3	36.7	11.1	0.4
**Mica:PVDF**	3.3	22.5	6.6	0.2
**Mica:30BT/PVDF**	5	141.8	27.4	3.9
**Mica:Kapton**	5	50.1	10.2	0.5
**Mica:PU**	>100	252.4	2.6	0.7

## References

[1] Li J., Zhang X., Ali S., Khan Z. (2020). Eco-innovation and energy productivity: New determinants of renewable energy consumption. J. Environ. Manag..

[2] Chen C., Pinar M., Stengos T. (2020). Renewable energy consumption and economic growth nexus: Evidence from a threshold model. Energy Policy.

[3] Chandrasekaran S., Bowen C., Roscow J., Zhang Y., Dang D.K., Kim E.J., Misra R.D.K., Deng L., Chung J.S., Hur S.H. (2019). Micro-scale to nano-scale generators for energy harvesting: Self powered piezoelectric, triboelectric and hybrid devices. Phys. Rep..

[4] Jiang D., Xu M., Dong M., Guo F., Liu X., Chen G., Wang Z.L. (2019). Water-solid triboelectric nanogenerators: An alternative means for harvesting hydropower. Renew. Sustain. Energy Rev..

[5] Luo J., Wang Z.L. (2019). Recent advances in triboelectric nanogenerator based self-charging power systems. Energy Storage Mater..

[6] Nozariasbmarz A., Collins H., Dsouza K., Polash M.H., Hosseini M., Hyland M., Liu J., Malhotra A., Ortiz F.M., Mohaddes F. (2020). Review of wearable thermoelectric energy harvesting: From body temperature to electronic systems. Appl. Energy.

[7] Costa P., Nunes-Pereira J., Pereira N., Castro N., Gonçalves S., Lanceros-Mendez S. (2019). Recent Progress on Piezoelectric, Pyroelectric, and Magnetoelectric Polymer-Based Energy-Harvesting Devices. Energy Technol..

[8] Lu Y., O’Riordan E., Cottone F., Boisseau S., Galayko D., Blokhina E., Marty F., Basset P. (2016). A batch-fabricated electret-biased wideband MEMS vibration energy harvester with frequency-up conversion behavior powering a UHF wireless sensor node. J. Micromech. Microeng..

[9] Chen J., Wang Z.L. (2017). Reviving Vibration Energy Harvesting and Self-Powered Sensing by a Triboelectric Nanogenerator. Joule.

[10] Wang S., Lin L., Wang Z.L. (2015). Triboelectric nanogenerators as self-powered active sensors. Nano Energy.

[11] Wang Z.L. (2013). Triboelectric Nanogenerators as New Energy Technology for Self-Powered Systems and as Active Mechanical and Chemical Sensors. ACS Nano.

[12] Kim D.W., Lee J.H., Kim J.K., Jeong U. (2020). Material aspects of triboelectric energy generation and sensors. NPG Asia Mater..

[13] Wang Z.L. (2017). On Maxwell’s displacement current for energy and sensors: The origin of nanogenerators. Mater. Today.

[14] Dharmasena G.R.D.I., Silva S.R.P. (2019). Towards optimized triboelectric nanogenerators. Nano Energy.

[15] Saxon D.J., Luke A.M., Sajjad H., Tolman W.B., Reineke T.M. (2020). Next-generation polymers: Isosorbide as a renewable alternative. Prog. Polym. Sci..

[16] Yoon J.H., Ryu H., Kim S.-W. (2018). Sustainable powering triboelectric nanogenerators: Approaches and the path towards efficient use. Nano Energy.

[17] Wang Z.L. (2016). Triboelectrification, in Triboelectric Nanogenerators.

[18] Pan S., Zhang Z. (2019). Fundamental theories and basic principles of triboelectric effect: A review. Friction.

[19] McCarty S.L., Whitesides G.M. (2008). Electrostatic Charging Due to Separation of Ions at Interfaces: Contact Electrification of Ionic Electrets. Angew. Chem. Int. Ed..

[20] Friedrich K. (2018). Polymer composites for tribological applications. Adv. Ind. Eng. Polym. Res..

[21] Lee J.H., Kim J., Kim T.Y., Al Hossain M.S., Kim S.W., Kim J.H. (2016). All-in-one energy harvesting and storage devices. J. Mater. Chem. A.

[22] Wang Z.L., Lin L., Chen J., Niu S., Zi Y., Wang Z., Lin L., Chen J., Niu S., Zi Y. (2016). Triboelectric Nanogenerator: Vertical Contact-Separation Mode. Triboelectric Nanogenerators.

[23] Marinho T., Costa P., Lizundia E., Costa C.M., Corona-Galván S., Lanceros-Méndez S. (2019). Ceramic nanoparticles and carbon nanotubes reinforced thermoplastic materials for piezocapacitive sensing applications. Compos. Sci. Technol..

[24] Martins P., Lopes A.C., Lanceros-Mendez S. (2014). Electroactive phases of poly(vinylidene fluoride): Determination, processing and applications. Prog. Polym. Sci..

[25] Ribeiro C., Costa C.M., Correia D.M., Nunes-Pereira J., Oliveira J., Martins P., Goncalves R., Cardoso V.F., Lanceros-Mendez S. (2018). Electroactive poly(vinylidene fluoride)-based structures for advanced applications. Nat. Protoc..

[26] Costa P., Silva J., Mendez S.L. (2016). Strong increase of the dielectric response of carbon nanotube/poly(vinylidene fluoride) composites induced by carbon nanotube type and pre-treatment. Compos. Part B Eng..

[27] Wang Z.L. (2016). Triboelectric Nanogenerators.

[28] Diaz F.A., Felix-Navarro R.M. (2004). A semi-quantitative tribo-electric series for polymeric materials: The influence of chemical structure and properties. J. Electrost..

[29] Lapčinskis L., Mālnieks K., Linarts A., Blūms J., Šmits K., Järvekülg M., Knite M., Šutka A. (2019). Hybrid Tribo-Piezo-Electric Nanogenerator with Unprecedented Performance Based on Ferroelectric Composite Contacting Layers. ACS Appl. Energy Mater..

[30] Zhang J.H., Li Y., Du J., Hao X., Huang H. (2019). A high-power wearable triboelectric nanogenerator prepared from self-assembled electrospun poly(vinylidene fluoride) fibers with a heart-like structure. J. Mater. Chem. A.

[31] Singh H.H., Khare N. (2018). Flexible ZnO-PVDF/PTFE based piezo-tribo hybrid nanogenerator. Nano Energy.

[32] Cheng X., Tang W., Song Y., Chen H., Zhang H., Wang Z.L. (2019). Power management and effective energy storage of pulsed output from triboelectric nanogenerator. Nano Energy.

[33] Zhang X.S., Han M., Kim B., Bao J.F., Brugger J., Zhang H. (2018). All-in-one self-powered flexible microsystems based on triboelectric nanogenerators. Nano Energy.

[34] Zou H., Zhang Y., Guo L., Wang P., He X., Dai G., Zheng H., Chen C., Wang A.C., Xu C. (2019). Quantifying the triboelectric series. Nat. Commun..

[35] Neagoe M.B., Prawatya Y.E., Zeghloul T., Dascalescu L. (2016). Influence of surface roughness on the tribo-electric process for a sliding contact between polymeric plate materials. IOP Conference Series: Materials Science and Engineering, Proceedings of the 13th International Conference on Tribology, Galati, Romania, 22–24 September 2016.

[36] Cheng G.G., Jiang S.Y., Li K., Zhang Z.Q., Wang Y., Yuan N.Y., Ding J.N., Zhang W. (2017). Effect of argon plasma treatment on the output performance of triboelectric nanogenerator. Appl. Surf. Sci..

[37] Helseth L.E. (2019). The Influence of Microscale Surface Roughness on Water-Droplet Contact Electrification. Langmuir.

[38] Shao Y., Feng C.P., Deng B.W., Yin B., Yang M.B. (2019). Facile method to enhance output performance of bacterial cellulose nanofiber based triboelectric nanogenerator by controlling micro-nano structure and dielectric constant. Nano Energy.

[39] Araujo C.M., Costa C.M., Lanceros-Mendez S. (2014). Evaluation of dielectric models for ceramic/polymer composites: Effect of filler size and concentration. J. Non-Cryst. Solids.

[40] Mendes S.F., Costa C.M., Caparrós C., Sencadas V., Lanceros-Méndez S. (2012). Effect of filler size and concentration on the structure and properties of poly(vinylidene fluoride)/BaTiO_3_ nanocomposites. J. Mater. Sci..

[41] Lee L.H. (1994). Dual Mechanism for Metal-Polymer Contact Electrification. J. Electrost..

[42] Niu S., Wang S., Lin L., Liu Y., Zhou Y.S., Hu Y., Wang Z.L. (2013). Theoretical study of contact-mode triboelectric nanogenerators as an effective power source. Energy Environ. Sci..

